# Associations of biomarkers of neurodegenerative diseases with mild neurocognitive disorder in a cohort of patients with stable coronary heart disease

**DOI:** 10.1002/alz.095214

**Published:** 2025-01-09

**Authors:** Valerie Lohner, Laura Perna, Ben Schöttker, Robert Perneczky, Matthias Kliegel, Hermann Brenner, Ute Mons

**Affiliations:** ^1^ University of Cologne, Cologne Germany; ^2^ Max Planck Institute of Psychiatry, Munich, Bavaria Germany; ^3^ German Cancer Research Center, Heidelberg, Baden‐Wuerttemberg Germany; ^4^ LMU University Hospital, Munich Germany; ^5^ Université de Genève, Geneve, Geneve Switzerland

## Abstract

**Background:**

Coronary heart disease (CHD) is a well‐known risk factor for cognitive impairment and dementia, and blood biomarkers of neurodegenerative diseases may be utilised to identify people at higher risk of cognitive decline. Here, we aimed to investigate prospective associations between these biomarkers and mild neurocognitive disorder (MiND) after a follow‐up of ten years in patients with stable CHD, and potential effect modification by hypercholesterolemia and *ApoE* genotype.

**Method:**

Biomarkers of neurodegenerative diseases (glial fibrillary acidic protein (GFAP), neurofilament light chain (NfL), and phosphorylated tau181 (p‐tau181)) were measured in baseline blood serum samples using the Single‐Molecule Array (Simoa) Technology (Quanterix, USA) in a subset (n = 363) of a cohort of patients with stable CHD. MiND was defined as scores ≤ 21.8 on the Cognitive Telephone Screening Instrument (COGTEL). Hypercholesterolemia was categorised into none (normal total cholesterol (TC) levels without statin use), normal TC levels with statin use, or increased TC levels independent of statin use. We evaluated prospective associations of biomarkers of neurodegenerative disease with MiND using multivariable logistic regression models, adjusted for age, sex, study centre, hearing impairment, and comorbidities. We additionally checked for biomarker×*ApoE* genotype and biomarker×hypercholesterolemia interactions.

**Result:**

At follow‐up, 55 (15.2%) patients had developed MiND. Higher levels of NfL were associated with increased risks of developing MiND (OR (95%‐CI) per SD increase: 1.44 (1.01‐2.04)). Associations of p‐tau181 with MiND were depending on hypercholesterolemia, but not on *ApoE* genotype. Higher levels of p‐tau181 were associated with lower odds of developing MiND in patients without hypercholesterolemia (OR (95%‐CI) per SD increase: 0.09 (0.01‐0.39)) and higher odds of developing MiND in patients with increased TC levels (OR (95%‐CI) per SD increase: 7.83 (1.72‐103.20)), but not in patients with normal TC levels using statins. Levels of GFAP were not associated with MiND.

**Conclusion:**

Preliminary analyses suggest that NfL and p‐tau181 predict MiND after ten years in patients with stable CHD, and that the association of p‐tau181 with MiND was modified by hypercholesterolemia. This might imply that a deterioration in cognitive performance in this population might be halted through early management of hypercholesterolemia, however, more research is warranted.

